# Development and Characterization of a Gelatin-Based Photoactive Hydrogel for Biomedical Application

**DOI:** 10.3390/jfb16020043

**Published:** 2025-01-29

**Authors:** Antanas Straksys, Adei Abouhagger, Monika Kirsnytė-Šniokė, Tatjana Kavleiskaja, Arunas Stirke, Wanessa C. M. A. Melo

**Affiliations:** 1 Department of Functional Materials and Electronics, State Research Institute Center for Physical Sciences and Technology (FTMC), LT-02300 Vilnius, Lithuania; antanas.straksys@ftmc.lt (A.S.); adei.abouhagger@ftmc.lt (A.A.); monika.kirsnyte@ftmc.lt (M.K.-Š.); arunas.stirke@ftmc.lt (A.S.); 2 Department of Polymer Chemistry, Institute of Chemistry, Vilnius University, LT-03225 Vilnius, Lithuania; tatjana.krivorotova@chf.vu.lt

**Keywords:** hydrogel, methylene blue, photodynamic therapy, gel-like, bacteria, fungi

## Abstract

Photoactive hydrogels facilitate light-triggered photochemical processes, positioning them as innovative solutions in biomedical applications, especially in antimicrobial photodynamic therapy. This study presents a novel methylene blue-based photoactive hydrogel designed as a topical gel solution to overcome the limitations of traditional pad-based systems by offering enhanced adaptability to irregular wound surfaces, uniform photosensitizer distribution, and deeper therapeutic light penetration. This study investigated the development of hydrogels by cross-linking gelatin with glutaraldehyde (GA) and incorporating methylene blue (MB) to investigate the effects of cross-linking density, network structure, and small molecule inclusion on hydrogel properties. The results showed that while glutaraldehyde concentration influenced swelling behavior and network structure, the inclusion of MB altered these properties, particularly reducing swelling and MB retention at higher GA concentrations. Rheological and thermal analyses confirmed that higher GA concentrations made the hydrogels more rigid, with MB influencing both mechanical and thermal properties. Additionally, the hydrogels exhibited enhanced antimicrobial properties through increased reactive oxygen species production, particularly in light-activated conditions, demonstrating the potential of MB-based photoactive hydrogels for improving antimicrobial efficacy, especially against *S. aureus*, *E. coli*, and *C. albicans*, offering as a possible alternative to traditional antimicrobial treatments.

## 1. Introduction

Photoactive hydrogels have garnered significant attention in biomedical research due to their ability to facilitate light-induced photochemical reactions for therapeutic applications. Methylene blue (MB), a cationic thiazine dye, is particularly notable for its capacity to generate reactive oxygen species (ROS) upon light activation, making it effective in antimicrobial photodynamic therapy (aPDT) for wound healing [1]. The generation of ROS at the wound site can eradicate pathogenic microbes while minimizing damage to surrounding healthy tissue [2]. However, the efficacy of these hydrogels is often influenced by their structural design and the stability of the photosensitizer within the system [3].

Traditional photoactive hydrogels, typically presented as preformed pads or sheets, encounter several limitations in clinical applications. These include inadequate flexibility to conform to irregular wound surfaces, uneven photosensitizer distribution, and challenges in ensuring sustained therapeutic effects [4]. Moreover, pads often restrict the ability to treat wounds of varying shapes and depths, limiting their practicality [5]. To address these issues, there is a growing interest in developing hydrogels as topical gel solutions. Such formulations offer enhanced adaptability, ease of application, and consistent coverage, especially for complex wound geometries [6].

This study introduces a novel MB-based photoactive hydrogel (HGMBs) designed explicitly as a topical gel solution rather than a pad. The hydrogel matrix is engineered to optimize the photochemical properties of MB, enhancing light absorption, ROS generation, and photochemical reactivity under near-infrared (NIR) light. The gel solution format allows for superior conformability to wound surfaces, ensuring uniform distribution of the photosensitizer and maximizing therapeutic efficacy [7].

By integrating advanced photochemical functionality with the flexibility of a gel solution, this hydrogel overcomes the limitations of traditional pad-based designs. The unique formulation enables deeper penetration of therapeutic light and a sustained release of ROS, ensuring effective antimicrobial activity and accelerated wound healing [4]. This dual focus on photochemical optimization and application flexibility marks a significant advancement in the design of photoactive hydrogels for biomedical use.

The present research offers an innovative approach to aPDT by developing an MB-based hydrogel as a topical gel solution, addressing mainly the photochemical limitations of existing systems. Thus, the study shows the photochemistry properties of MB-based photoactive hydrogel as well as the antimicrobial properties of it.

## 2. Materials and Methods

### 2.1. Materials Used for the Development of MB-Based Photoactive Hydrogel

Materials used included gelatin from porcine skin (gel strength ~175 Bloom, Type A), dimethyl formamide (analytical grade) from Sigma Aldrich, (St. Louis, MO, USA) glutaraldehyde (50%) from VWR Chemicals (Lutterworth, UK), methylene blue (3.7-bis(dimethylamino)-phenothiazin-5-ium chloride) from Alfa Aesar (Ward Hill, MA, USA), isopropanol (analytical grade) and ethylenediaminetetraacetic acid (EDTA) from the local company Eurochemicals (Vilnius, Lithuania), 1.3-diphenylisobenzofuran (DPIBF) from Thermo Scientific (Vilnius, Lithuania), and the enzyme *trypsin* (EC 3.4.24.4) from a porcine pancreas (1000–2000 BAEE units/mg, solid) from Sigma Aldrich (St. Louis, MO, USA).

### 2.2. Synthesis of MB-Based Photoactive Hydrogel

**Figure 1:**
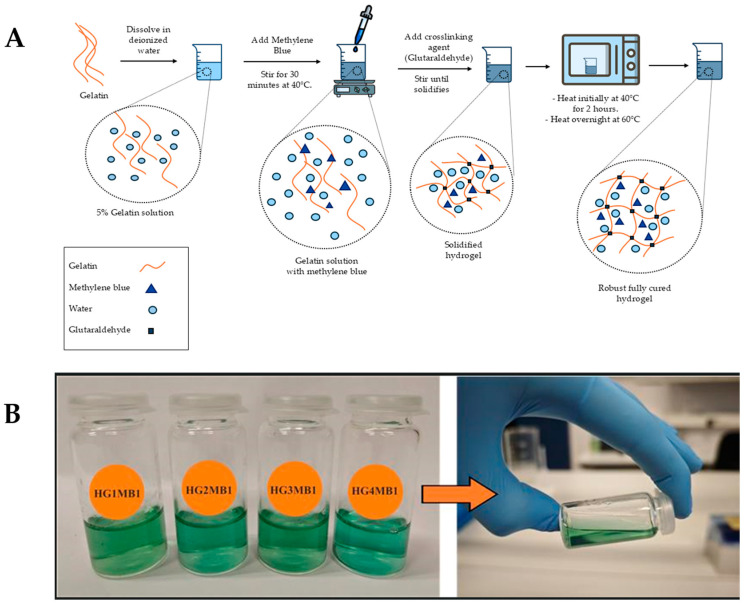
(**A**) Schematic representation of HGMBs synthesis, including preparation of gelatin solution, methylene blue incorporation, crosslinking with glutaraldehyde, and curing steps. (**B**) Photography of HGMBs and its gel solution form.

The synthesis of the MB-based photoactive hydrogel (HGMBs) and its gel-like morphology, resembling a liquid solution, are illustrated in Figure 1. Basically, its synthesis involves a series of carefully controlled steps to ensure a robust and homogeneous structure. First, a 5% gelatin solution was prepared by mixing gelatin with deionized water. Subsequently, methylene blue was incorporated at concentrations of 1.0 mg/mL. A magnetic stir bar was introduced, and the mixture was stirred for 30 min at 40 °C to ensure complete dissolution of both gelatin and methylene blue. Subsequently, a 25% glutaraldehyde (GA) solution was added to the beaker in volumes ranging from 20 to 80 µL/mL, and the solution was stirred continuously until it solidified. The GA was used as a crosslinking agent to stabilize the gelatin network, forming a robust hydrogel structure. The resulting hydrogel was then placed in a heating oven at 40 °C for 2 h to facilitate the crosslinking reaction, resulting in a homogeneous mass. After this initial period, the temperature was increased to 60 °C and maintained overnight to complete the curing process, during which the hydrogel was periodically mixed.

For hydrogels without methylene blue (HGs), deionized water was added at a 1:10 volume ratio, and the mixture was left to rest overnight to allow for complete swelling and to determine the true volume of the hydrogel. The mixture was then centrifuged at 2376 RCF for 10 min to remove excess water, which was decanted. In contrast, for HGMBs the step involving the addition of deionized water and overnight resting was omitted, as the methylene blue was already incorporated during the hydrogel preparation process.

The entire synthesis process of HGMBs was conducted under aluminum foil cover to prevent any exposure to light and avoid photobleaching of MB. Similarly, during heating in the oven, the samples were also covered with aluminum foil. All subsequent investigations of HGMBs were carried out with strict precautions to avoid exposure to both natural and artificial light.

### 2.3. Spectroscopic Measurements of MB-Based Photoactive Hydrogel

Fourier transform infrared (FT-IR) spectra of hydrogel with and without MB in dry film forms and primary reagents were recorded by Attenuated total reflectance (ATR) FT-IR Perkin Elmer FRONTIER spectrometer (London, UK). The FT-IR spectra were obtained under the following conditions: a scan range of 600–4000 cm^−1^, 20 scans, and a resolution of 2 cm^−1^.

### 2.4. Removal of Methylene Blue Excess from MB-Based Photoactive Hydrogel

The HGMBs were placed, separately, in a glasses beaker containing 50 mL of distilled water, and a magnetic stir bar was added to facilitate mixing. The beaker was then placed on a magnetic heating plate and heated at 40 °C for 1.5 h to facilitate initial mixing and equilibration. Following this heating step, the hydrogel solution was subjected to centrifugation at 2376 RCF for 10 min. This centrifugation step was crucial for removing excess MB in water from the hydrogel. The supernatant was decanted from the hydrogel and again refilled with deionized water. The washing process, including the heating and centrifugation steps, was repeated until the deionized water no longer exhibited any detectable absorption of MB, indicating that the PHB had been adequately purified.

### 2.5. MB-Based Photoactive Hydrogel Swelling Kinetic Test

A pre-weighed portion of the prepared hydrogel (with or without MB) was placed into a glass beaker and dried in an oven at 80 °C until a constant mass was achieved. The swelling ratio (SW, %) was subsequently calculated using the following formula:

(1) $$\mathrm{SW}\%=\frac{w_{s}-w_{d}}{w_{d}}\cdot 100$$

Calculation of M_c_ (number of average molecular weight of chain segments between cross-linking points) and v_e_ (the cross-link density in terms of the number of elastically effective chains per unit volume of the gelatin hydrogel network) was performed following the methodology outlined by Rattanaruengsrikul et al. [8]:

(2) $$v_{G}=\frac{W_{d}\cdot \rho_{w}}{\left[\mathrm{Ws}-W_{d}\cdot \left(\rho_{G}-\rho_{W}\right)\right]}$$

(3) $$M_{c}=\frac{-\rho_{G}\cdot V_{1}\cdot v_{G}^{1/3}}{\left[\left(X\cdot v_{G}^{2}/2\right)+\ln\left(1-v_{G}\right)+v_{G}\right]}$$

(4) $$V_{e}=\frac{\rho_{G}\cdot {\;N}_{A}}{M_{c}};$$

where:

W_d_—dry hydrogel mass;
W_s_—swelled hydrogel mass;
ρ_G_—gelatin density (1.35 g/mL);
ρ_w_—water density at 25 °C (0.997 g/mL);
V_1_—molar water volume (18.07 mL/mol);
V_G_—gelatin mass portion in swelled hydrogel;
X—the Flory–Huggins interaction (0.49 ± 0.05);
N_A_—Avogadro’s number (6.022 × 10^23^).

### 2.6. Rheological Analysis of MB-Based Photoactive Hydrogel

Rheological analysis of the HGMBs was conducted following the procedure outlined in [9]. Briefly, rheological measurements were performed using a rotational rheometer (CR302, Anton Paar Ltd., Graz, Austria) equipped with a parallel plate system consisting of a round disc with a 0.5 mm gap and a 5.0 mm plate diameter. To determine the gelation temperature, the storage modulus (G′) and loss modulus (G′′) were measured during a temperature sweep from 20 to 50 °C at a rate of 1 °C/min. The angular frequency and strain amplitude were kept constant at 1 Hz and 0.1%, respectively. Additionally, rotational tests were conducted at a shear rate ranging from 0.1 s^−1^ to 100 s^−1^ at a constant temperature of 37 °C. Frequency sweep analysis within the linear viscoelastic region was performed at 37 °C across a broad oscillation frequency range of 0.1 to 100 rad/s to assess the dynamic viscoelastic properties of the HGMBs.

The crosslinking density of soft hydrogel, named ne (mol/cm^3^), was calculated from the plateau value of storage modulus (G′), which was measured across a temperature range (20–50 °C) and taken values at 37 °C, according to methods [10]:

(5) $$n_{e}=\frac{\;G\;\prime }{R\cdot T}$$

where:

G′—storage modulus, Pa

R—universal gas constant 8.3144 Pa ∗ m^3^/(mol ∗ K)

T—absolute temperature, K.

### 2.7. Quantification of Methylene Blue Content in MB-Based Photoactive Hydrogel

A 0.5 g sample of HGMBs was placed in a glass beaker and mixed with 10 mL of PBS buffer containing 0.25% trypsin (Sigma-Aldrich, St. Louis, MO, USA), an enzyme capable of digesting the gelatin matrix of the hydrogel. The mixture was incubated at 37 °C overnight to facilitate enzymatic digestion. Following incubation, the mixture was heated at 90 °C for 30 min to inactivate the enzyme. The absorbance of the solution was then measured at 610 nm using a UV-Vis spectrophotometer to determine the amount of MB present in the HGMBs samples. For comparison, a control was prepared using hydrogel HGs samples, which are without MB.

### 2.8. Differential Scanning Calorimetry Analysis of MB-Based Photoactive Hydrogel

Differential scanning calorimetry (DSC) analysis was conducted to evaluate the thermal properties of gelatin and the synthesized hydrogel. Prior to analysis, the samples were lyophilized to remove moisture, ensuring accurate thermal measurements. The lyophilized samples were weighed and sealed in aluminum pans, while an empty pan served as the reference. The DSC measurements were performed under a nitrogen atmosphere to prevent oxidative degradation, with a heating rate of 10 °C/min over a temperature range of −50 °C to 250 °C. The resulting DSC curves were used to determine the glass transition temperature (Tg) and other thermal characteristics of the materials.

### 2.9. Assessment of ROS Generation by MB-Based Photoactive Hydrogel

The ROS generation by the HGMBs was evaluated following the method of J. Černý et al. (2010) [11], with minor modifications. The ROS test was conducted in a 40% DMF (dimethylformamide) solution to evaluate the stability of DPIBF (1,3-diphenylisobenzofuran) under these conditions, with calibration achieved using a correlation coefficient of R^2^ = 0.996. ROS was generated from hydrogel following irradiation with a red LED light source designed and manufactured by FTMC. The LED device operated at a predominant wavelength of 630 nm, with a tip diameter of 11 mm with an 18 mW/cm^2^ light intensity delivered to the sample. The test system consisted of dissolving MB and DPIBF in anhydrous DMF. A control sample was prepared using 40% DMF without MB and exposed to red light, while an additional control containing MB and DPIBF in 40% DMF was kept in the dark. These controls did not show ROS generation, establishing as a baseline for ROS detection.

For ROS generation, MB was used as a photosensitizer, leading to the subsequent cleavage of DPIBF upon light exposure. The experiment duration was 30 min, during which DPIBF was fully consumed. The absorbance of MB at 410 nm was approximately 0.01 OD, while the initial OD for the DPIBF and MB system was set at 1.5.

In a separate setup, a specific amount of swollen hydrogel was placed into a glass vial, which was then filled with DPIBF in a 40% DMF solution, ensuring that the overall OD did not exceed 1.5. The vial was thoroughly mixed and then exposed to red light (λ = 630 nm) for 30 min. Post-irradiation, the absorbance of the solution was measured at 410 nm using a UV-Vis spectrophotometer (Halo RB-10, Dynamica, Graz, Austria) to assess the remaining DPIBF concentration. The amount of ROS generated was calculated using the following formula:

(6) ROS (mol)=initial DPIBF (mol)−measurment DPIBF(mol)

### 2.10. Antimicrobial Properties of MB-Based Photoactive Hydrogel

#### 2.10.1. Strains and Culture Conditions

*Escherichia coli* (K12, DMS498), *Staphylococcus aureus* (NCTC 11963), and *Candida albicans* (ATCC 10231) were used to verify the antimicrobial effect of the MB-based photoactive hydrogel. *E. coli* and *S. aureus* were grown aerobically in brain–heart infusion (BHI-broth) (Oxoid, London, UK) at 37 °C and 150 rpm, until logarithm phase. *C. albicans* was grown aerobically in Yeast (YPD-broth) (Oxoid, UK) at 37 °C and 150 rpm. The microorganisms were harvested by centrifugation (5 min, 5000 rpm) and resuspended in sterile phosphate buffered saline (PBS) at pH 7.0 to final concentration of 1 × 10^7^ cell mL^−1^.

#### 2.10.2. aPDT Procedure

A total of 100 μL of the microorganisms’ suspensions (10^7^ cell mL^−1^) were transferred into 96 wells-microtiter plate. Identical-volume MB-based photoactive hydrogel (HG1MB1) was added to each well. After the incubation time of 30 min at 37 °C in the dark, the samples were irradiated at 630 nm with a light dose of 24 J cm^−2^. Cell viability was determined by using the micro drop technique and the colony counting was performed after 24 h of incubation at 37 °C to calculate the Colony-forming unit (CFU).

Control samples were added to compare with the effectiveness of MB- photoactive hydrogel. These were named as follows: Control (L−)—only microorganism without irradiation; Control (L+)—only microorganism with irradiation; MB (L−)—methylene blue (1 mg/mL) without irradiation; MB (L+)—methylene blue (1 mg/mL) with irradiation; and HG1MB1 (L−)—without irradiation.

### 2.11. Statistical Analysis

Values are given as means and standard errors of four separate experiments. Differences between HGMBs, HGs, and MB were tested for significance via one way ANOVA with Tukey post-hoc test, mainly for antimicrobial analysis. *p*-values of less than 0.05 were considered significant.

## 3. Results

MB-based photoactive hydrogels and hydrogels without MB were synthesized from gelatin with the addition of 20–80 µL/mL of 25% of GA as a crosslinking agent, effectively transforming the soluble gelatin into a stable, soft hydrogel matrix. Based on the concentration of GA used (Table 1), the resulting hydrogels without MB were designated as HG1, HG2, HG3, and HG4. Additionally, MB was incorporated into the hydrogel concentration of 1 mg/mL, and these samples were labeled as HG1MB1, HG2MB1, HG3MB1, and HG4MB1, respectively.

Following synthesis, key physical properties of the hydrogels, including swelling ratio and average molecular weight of chain segments between cross-linking points (*M_c_*) and the cross-linking density (*V_e_*), were determined. Viscosimetric analysis was also conducted for HGMBs to further characterize their structural properties.

### 3.1. Fourier Transform Infrared (FT-IR) Spectroscopy

**Figure 2:**
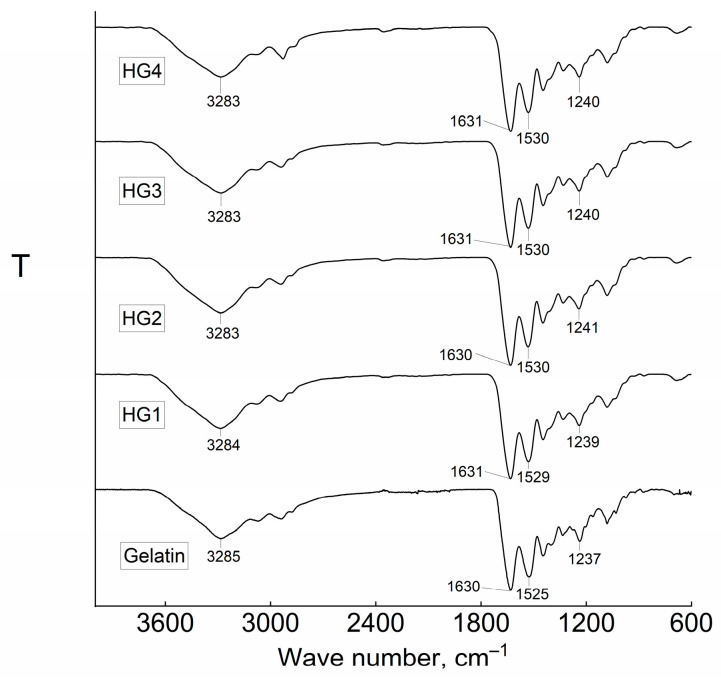
FT-IR spectra of gelatin and HGs.

**Figure 3:**
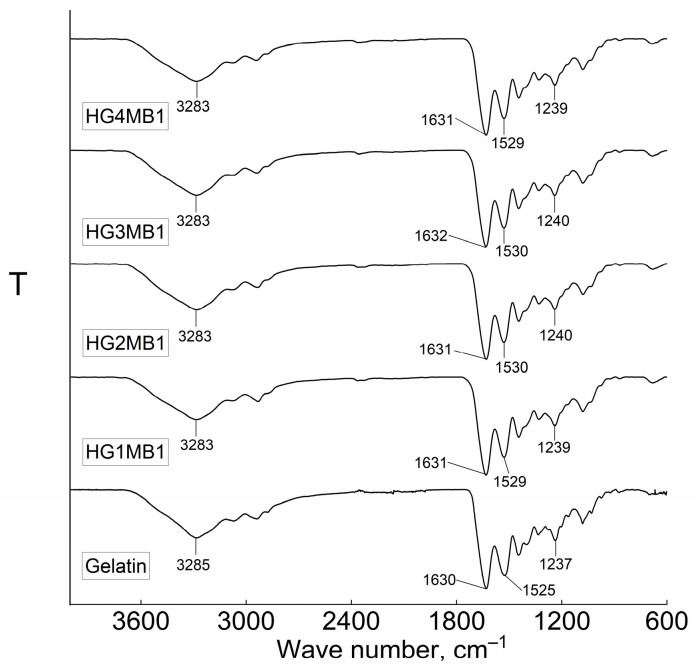
FT-IR spectra of gelatin and HGMBs.

The FT-IR spectra, shown in Figure 2 and Figure 3, provide insights into the chemical structure of HGs and HGMBs. The spectrum for HGs exhibited characteristic absorption bands at 3285 cm⁻^1^, corresponding to the stretching vibrations of N–H and O–H bonds (amine and hydroxyl groups), at 1630 cm⁻^1^ for the amide I band (C=O and C=N stretching vibrations), at 1525 cm⁻^1^ for the amide II band (N–H bending and C–N stretching vibrations), and at 1237 cm^−1^ the amide III (CN stretching and NH bending vibrations) [12]. After cross-linking gelatin with GA at concentrations of 20–80 µL/mL, slight shifts in these characteristic bands were observed. Specifically, the hydroxyl and amine band shifted from 3285 to 3283 cm⁻^1^, the amide I band moved to 1631–1630 cm⁻^1^, the amide II band shifted to 1527–1529 cm⁻^1^, and the amide III moved to 1241–1240 cm^−1^, respectively (Figure 2). These shifts are attributed to the formation of Schiff bases between the amine groups of gelatins with the aldehyde groups of GA, as reported in the literature [13,14]. Also, the excess GA could initiate the formation of enamine groups as a secondary reaction between secondary amines and aldehyde groups of GA.

An interesting observation was made when MB was incorporated into the synthesis. In HGMBs, the amine or hydroxyl bands appeared at the same position, 3283 cm⁻^1^, as in HGs, but it still differed from that of pure gelatin. The amide I band of HGMBs slightly shifted to 1631–1632 cm⁻^1^, while the amide II band remained unchanged at 1530–1529 cm⁻^1^, consistent with HGs. Additionally, the amide III bands shifted to 1240–1239 cm⁻^1^ (Figure 3).

FT-IR data reveal that the dependence of the synthesis pathway on GA concentration is complex. During synthesis, new bonds such as imines and enamines are formed, generating peaks at positions coinciding with gelatin amide bonds. However, shifts in the amide (I, II, and III) band peaks confirm the synthesis. Additionally, the use of a diluted solution during synthesis resulted in a low cross-linking density of the soft HGMBs. These observations suggest that MB played a role in the formation of HGMBs, potentially influencing the formation of Schiff bases between gelatin and GA.

### 3.2. Swelling Kinetics of MB-Based Photoactive Hydrogel

The cross-linking of gelatin using GA successfully transformed the material into a hydrogel with high water-insolubility and substantial swelling capacity. The swelling ratios for hydrogels HG1–HG4 ranged from 8599% to 9067% (Table 1), indicating high water absorption due to the low gelatin concentration in the preparation solution. However, the lack of a consistent trend with increasing GA concentration suggests that other factors, such as network heterogeneity or preparation conditions, may influence swelling behavior. The calculated molecular weight between cross-linking points (M_c_) and cross-linking density (V_e_) were relatively stable (2.7–2.9 × 10^+5^ g/mol and 2.8–3.1 × 10^+18^ cross-links/cm^3^, respectively), suggesting that GA concentration had a limited impact on the network structure in the absence of MB.

The results demonstrate that MB actively participates in the cross-linking reaction between gelatin and GA, influencing the hydrogel’s structural properties. As the ratio of GA increased, the swelling ratio of the hydrogels decreased, reflecting the impact of cross-linking density on hydrogel behavior. Specifically, the swelling ratio of HG1MB1 was 53,581%, while the intermediate GA concentration in HG3MB1 resulted in a decrease to 44,115%. At the highest GA concentration (HG4MB1), the swelling ratio further decreased to 36,425%. This trend indicates a complex relationship between cross-linking density and hydrogel structure, suggesting that increased GA concentrations lead to a more compact network, reducing the hydrogel’s ability to absorb water.

When comparing HGs to HGMBs, the swelling ratio increased by four to six times, highlighting that MB has a significant effect on the hydrogel matrix formation and cross-linking behavior. This suggests that MB not only modifies the physical properties of the hydrogel but also plays a crucial role in its synthesis.

In contrast to previous studies on gelatin-GA hydrogels [15], which did not specify whether MB was incorporated during the synthesis or added post-synthesis, our study provides direct evidence that MB participates in the cross-linking process. These earlier studies also did not explore the interactions between MB and GA during synthesis, leaving a critical gap in understanding the role of MB in hydrogel formation. However, by comparing hydrogels with and without MB, we show that MB incorporation significantly affects key characteristics, including swelling behavior and viscoelasticity. Specifically, MB contributes to increased crosslinking density, leading to reduced swelling and enhanced mechanical rigidity. These findings provide deeper insights into MB’s role in gelatin-based hydrogels and underscore its functional significance in hydrogel design for applications beyond controlled release systems.

### 3.3. Retention and Quantification of Methylene Blue in MB-Based Photoactive Hydrogel

After synthesizing HGMBs, the excess of MB was removed by washing the hydrogels with deionized water after twice soaking in phosphate-buffered saline (PBS) solution due to the change. The final MB content in the hydrogels was determined using enzymatic hydrolysis with trypsin, which cleaves proteins into amino acids, fully decomposing the HGMBs. The absorbance of MB in the solution was measured, and the MB content was calculated. Results indicate that the amount of MB retained in the hydrogels decreased with increasing cross-linking agent concentration. Specifically, increasing the GA concentration from 20 to 80 µL/mL reduced the MB content in hydrogel from 0.416 mg/g to 0.298 mg/g (Table 1).

### 3.4. Rheological Properties of the MB-Based Photoactive Hydrogel

**Figure 4:**
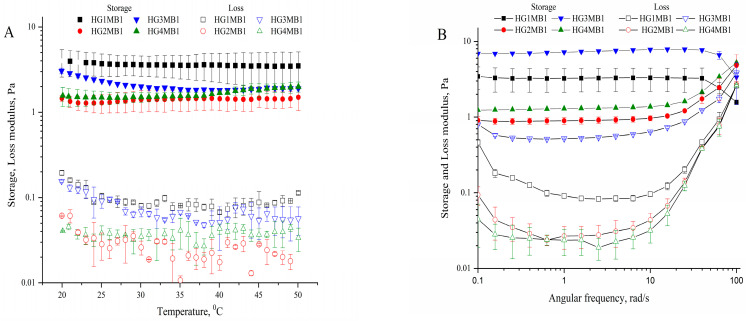
(**A**) Storage and loss moduli of HGMBs across temperature range (20–50 °C) and (**B**) frequency dependence.

The storage modulus (G′) and loss modulus (G′′) of the hydrogels were measured over a temperature range of 20–50 °C at a frequency of 1 Hz and a strain of 0.1% (Figure 4A). Across all samples, the storage modulus (G′) was consistently at least 30 times higher than the loss modulus (G′′), confirming the predominantly elastic behavior of the hydrogels. This significant difference indicates the strong cross-linked network within the hydrogel matrix, ensuring structural stability under thermal variations.

The elastic, gel-like behavior of the HGMBs remained stable across the entire temperature range, with only minor variations in G′. This stability suggests that the cross-linking density provided by GA and the inclusion of MB produce thermally robust hydrogels suitable for biomedical applications. While G′′ exhibited slight increases with rising temperature, its overall lower values compared to G′ highlight minimal viscous dissipation even at elevated temperatures. Notably, hydrogels with higher GA concentrations (e.g., HG4MB1) showed slightly reduced G′ values compared to those with lower GA concentrations (e.g., HG1MB1), suggesting that increasing cross-linking density may affect the flexibility of the hydrogel matrix.

The viscoelastic properties of the hydrogels were additionally assessed through frequency sweep analysis, revealing distinct trends in the storage modulus (G′) and loss modulus (G′′) as a function of angular frequency (Figure 4B). Across all samples, G′ remained consistently higher than G′′ over the entire frequency range, indicating that the hydrogels exhibited predominantly elastic behavior. G′ showed minimal variation at lower frequencies (<1 rad/s), reflecting the robust structural integrity of the hydrogels. However, at higher frequencies (>10 rad/s), G′ increased slightly, suggesting enhanced stiffness under rapid deformation. In contrast, G′′, which represents viscous dissipation, remained significantly lower than G′, with a slight increase at higher frequencies, indicating some viscous response under dynamic conditions. Notably, hydrogels with lower GA concentrations (e.g., HG1MB1) displayed higher G′ values compared to those with higher GA concentrations (e.g., HG4MB1), underscoring the impact of cross-linking density on the elastic properties. These results highlight that increasing GA concentrations create denser, more rigid hydrogel networks, reducing their flexibility and storage capacity.

**Figure 5:**
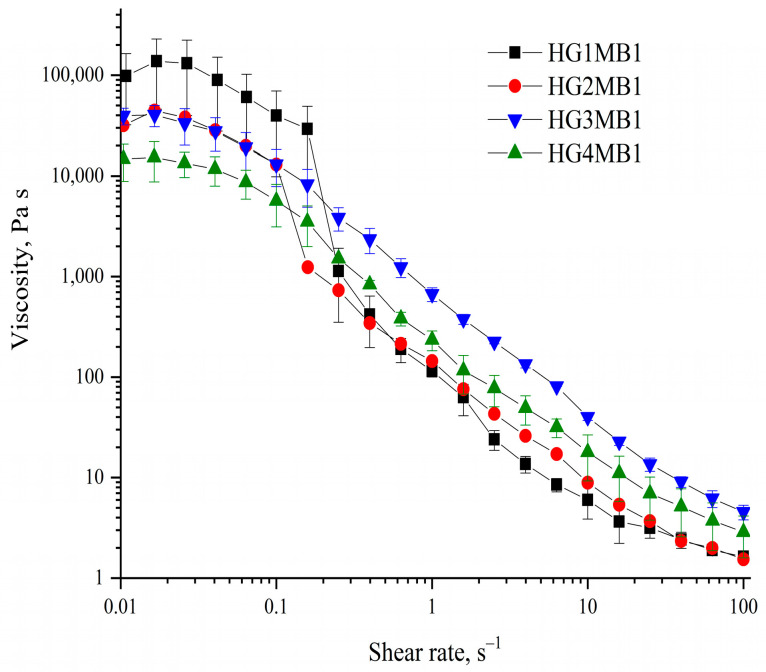
Viscosity profiles of HGMBs as a function of shear rate (0.01–100 s^−1^).

The flow behavior of the HGMBs was evaluated through viscosity measurements as a function of shear rate, providing insights into their non-Newtonian characteristics and the influence of cross-linking density on rheological properties. All HGMBs exhibited non-Newtonian shear-thinning behavior over the shear rate range of 0.01 to 100 s⁻^1^, as shown in Figure 5. HG1MB1 and HG2MB1 displayed significantly higher viscosities compared to HG3MB1 and HG4MB1, particularly at lower shear rates. For HG1MB1, viscosity decreased markedly from approximately 150,000 Pa·s at 0.01 s⁻^1^ to 120 Pa·s at 100 s⁻^1^, reflecting a reduction of over 1250 times. Similarly, HG2MB1 showed a sharp decrease in viscosity, from around 48,000 Pa·s to 140 Pa·s, corresponding to a 340-fold reduction. In contrast, HG3MB1 and HG4MB1 exhibited less pronounced viscosity decreases of approximately 53 and 72 times, respectively, across the same shear range.

The variations in viscosity and shear-thinning behavior indicate that the degree of cross-linking, governed by GA concentration, plays a significant role in determining the rheological properties of the hydrogels. Furthermore, these changes in viscoelastic behavior may be attributed to reduced electrostatic interactions between gelatin chains as chemical cross-linking increases, consistent with findings from previous studies [16,17].

### 3.5. Cross-Linking Density Trends Across Swelling and Rheological Methods

The cross-linking density (Ve) of the HGMBs (Table 2) was derived from swelling data, while the cross-linking density (ne) was calculated from rheological data using the storage modulus (G′) presented in Figure 4A at 37 °C.

The results indicate that the V_e_ values are influenced by GA concentration, increasing from 2.41 × 10^7^ to 4.58 × 10^7^ mol/cm^3^. In contrast, the cross-linking density (n_e_), determined from rheological data, ranged from 1.38 × 10^9^ to 6.01 × 10^10^ mol/cm^3^. The discrepancy between V_e_ and n_e_ can be attributed to the non-invasive nature of the swelling method, which does not involve the application of forces, spinning, or rotation. Conversely, the synthesized HGMBs are very soft, with a low cross-linking density and a high capacity for water absorption. Consequently, during rheological measurements, the applied forces may compress the HGMBs, causing a reduction in its initial volume and expansion. This compression results in lower ne values compared to V_e_.

### 3.6. Differential Scanning Calorimetry Analysis of MB-Based Photoactive Hydrogel

**Figure 6:**
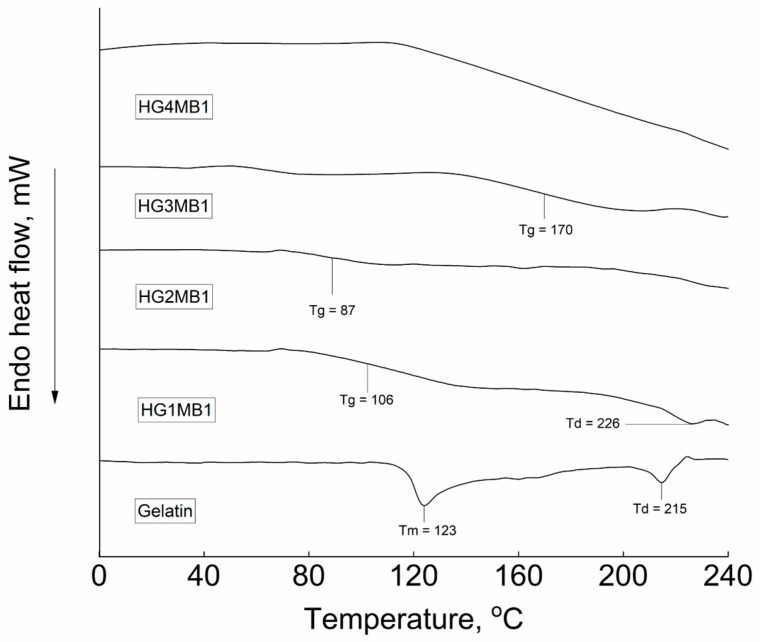
Differential scanning calorimetry curves of gelatin and HGMBs.

The thermogram of gelatin (Figure 6) displayed typical phase transitions, including a melting temperature (Tm) of 123 °C and an isomerization temperature (Td) of 215 °C [18]. In contrast, the thermal properties of the synthesized HGMB hydrogel exhibited distinct behavior, which may be influenced by the concentration of glutaraldehyde (GA). The thermogram of HG1MB1, crosslinked with the lowest GA concentration, displayed a glass transition temperature (Tg) of 106 °C and an absence of the melting transition (Tm) typically observed for gelatin. Additionally, HG1MB1 exhibited a slightly elevated degradation temperature (Td) of 226 °C, as observed during the thermal analysis. The thermograms of HG2MB1, HG3MB1, and HG4MB1 were similar, lacking significant endothermic transitions. However, in the cases of HG2MB1 and HG3MB1, the thermograms showed a poorly defined glass transition at 87 °C and 170 °C, respectively. As the concentration of GA increased during the crosslinking process, the hydrogels exhibited an increase in covalent bonding, leading to a more rigid amorphous structure. In the case of HG4MB1, no clear transitions were observed, likely due to the high concentration of crosslinking bonds.

### 3.7. ROS Quantification

The quantity of ROS generated by hydrogels with incorporated MB was determined following a method described in the literature [11], with minor modifications. The methods and corresponding results are summarized in Table 1, highlighting the relationship between cross-linking density and ROS production. For the ROS assay, 1,3-diphenylisobenzofuran (DPIBF) was employed as a singlet oxygen scavenger. Upon reaction with ROS, DPIBF undergoes a chemical transformation, resulting in a loss of its characteristic color. The results indicated that the hydrogel HG1MB1, which contained the highest amount of MB, generated the highest amount of ROS (1.7 ×·10^−4^ mol/g). HG2MB1, HG3MB1 and HG4MB1 produced 1.5 ×·10^−4^, 1.4 ×·10^−4^, and 1.1 ×·10^−4^ mol/g of ROS, respectively. Despite slight variations, the hydrogels with MB produced similar levels of ROS, suggesting that they could be utilized as antimicrobial hydrogels.

### 3.8. Antimicrobial Properties of MB-Based Photoactive Hydrogel

In this experiment, only HG1MB1 was applied as it demonstrated superior photochemical results, including reactive oxygen species (ROS) production, compared to the other HGMBs used in this study.

**Figure 7:**
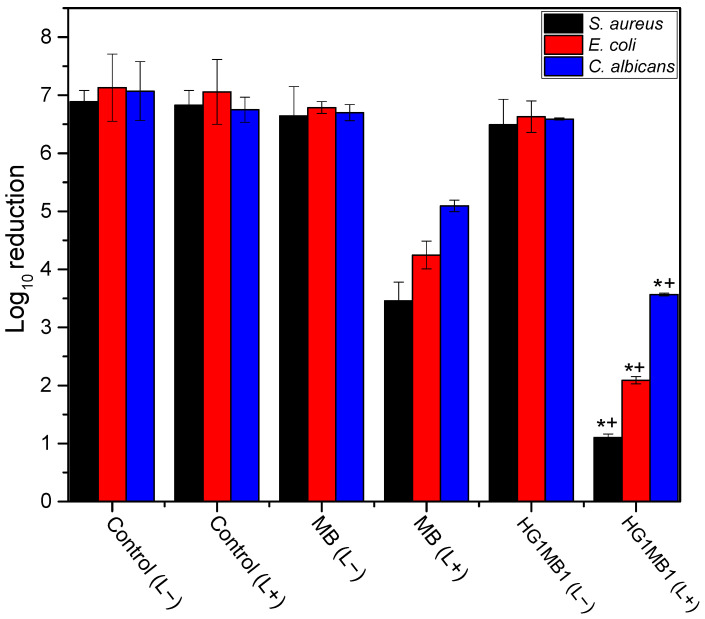
Log reduction of *S. aureus*, *E. coli*, and *C. albicans* after 30 min of incubation with MB-based photoactive hydrogel (HG1MB1) upon irradiation at 630 nm with a light dose of 24 J cm^−2^ and its respective controls. Values are the means of three separate experiments, and the bars are standard deviations. * *p* < 0.001 versus control (L+) and HG1MB1 (L−); † *p* < 0.01 versus MB (L+).

Based on the results presented in Figure 7, it can be inferred that the effect of MB following irradiation (630 nm—24 J/cm^2^) was enhanced when combined with the hydrogel, across all microorganisms tested in this study.

The control groups did not demonstrate a significant reduction in microorganism viability, particularly those without irradiation (L−). In contrast, the control MB (L+) exhibited a more substantial reduction in microorganisms compared to the control (MB (L−)), with a notable log reduction of 3.0 for *S. aureus*. Importantly, after aPDT treatment using HG1MB1, *S. aureus*, *E. coli*, and *C. albicans* showed significant inhibition, with log reductions of 5.40, 4.66, and 3.03, respectively, when compared to the untreated HG1MB1. These results are at least two times greater than those achieved using MB alone.

Comparing the aPDT results across microorganisms, it is evident that *S. aureus*, especially when treated with HG1MB1 (L+), experienced the greatest reduction after irradiation, followed by *E. coli* and *C. albicans*. While the differences between *E. coli* and *S. aureus*, as well as between *E. coli* and *C. albicans*, were not statistically significant, a significance level of *p* < 0.05 was observed when comparing *S. aureus* to *C. albicans* in the study with HG1MB1 (L+).

Thus, MB-based photoactive hydrogel shows the ability to inhibit different species of microorganism, improving the aPDT effect of MB.

## 4. Discussion

The development of hydrogels through the cross-linking of gelatin with GA, with and without the incorporation of MB, presents a comprehensive understanding of how cross-linking density, network structure, and small molecule incorporation impact hydrogel properties. Hydrogels, specifically HGMBs, are increasingly investigated for applications in drug delivery, wound healing, and antimicrobial systems due to their biocompatibility and tunable properties. The experiments conducted on these hydrogels provide valuable insights into their physical, mechanical, and chemical behavior, highlighting the role of MB in modifying the hydrogel structure.

The cross-linking of gelatin with varying concentrations of GA resulted in hydrogels (HG1 to HG4) that exhibited significant water absorption, as indicated by their high swelling ratios (8911% to 9067%). These results are consistent with prior studies on gelatin-based hydrogels, where the water uptake is directly influenced by the gelatin concentration and the cross-linking agent used [19]. However, the swelling ratios did not show a consistent trend with increasing GA concentration, which could be due to the influence of factors such as network heterogeneity or differences in the cross-linking efficiency under varying preparation conditions [20]. While GA concentration did not significantly alter the molecular weight between cross-linking points (M_c_) and cross-linking density (V_e_), these factors remained relatively stable across the hydrogels, suggesting that GA concentration only mildly influenced the network structure when MB was absent.

In contrast, when MB was incorporated into the hydrogels (HG1B1, HG2B1, HG3B1, HG4B1), the swelling behavior was significantly affected, indicating an interaction between MB, gelatin, and GA during the cross-linking process.

Specifically, the swelling ratio for the MB-containing hydrogels decreased as the GA concentration increased, suggesting that MB plays a role in modulating the hydrogel network. This phenomenon might be attributed to the ability of MB to interact with GA, influencing the degree of cross-linking and the available space for water uptake. The decrease in swelling at higher GA concentrations could also indicate that the increased cross-linking density limits the ability of the hydrogel to expand and absorb water [21]. These findings are consistent with those from other studies where small molecules or drugs incorporated into hydrogels affected their swelling properties [22].

The primary reaction between GA and gelatin occurs at basic amino acids, particularly lysine residues, forming imine bonds, also known as Schiff bases [23]. At higher GA concentrations, additional reactions with secondary amines may occur, leading to the formation of enamine bonds [15]. MB, as a positively charged dye, influences the synthesis pathway by electrostatically interacting with the negatively charged groups of acidic amino acids in gelatin [20,24]. Other interactions, such as van der Waals forces and hydrogen bonding, are also possible. For example, hydrogen bonds may form between the hydrogen of amino groups in basic amino acids and the nitrogen or sulfur in MB’s thiazine ring [20,25].

Under these conditions, MB may reduce GA’s reactivity with gelatin amino groups due to steric hindrance. At low GA concentrations, most MB is retained in the hydrogel. However, as GA concentration increases, competition between GA and MB reduces the blocking effect of MB, allowing GA to interact more freely with gelatin. Consequently, the swelling ratio of the hydrogel decreases with increasing GA concentration, and the amount of MB incorporated into the hydrogels declines.

This decline suggests that higher cross-linking densities result in fewer MB molecules being retained within the hydrogel matrix. The interaction between GA and MB likely confines MB within the dense network of gelatin chains, reducing its availability for release. This reduction in MB retention with increased cross-linking density aligns with previous findings, where enhanced cross-linking in hydrogels limited the encapsulation capacity of hydrophilic molecules [26].

Rheological measurements further elucidated the impact of cross-linking density on the mechanical properties of the hydrogels. The storage modulus (G′) was consistently much higher than the loss modulus (G′′), indicating that the hydrogels exhibited predominantly elastic behavior. This supports the hypothesis that the cross-linked networks formed by GA contribute to the hydrogel’s structural stability and elastic properties [27]. The trend observed in the rheological measurements, where hydrogels with lower GA concentrations (e.g., HG1MB1) had higher G′ values than those with higher GA concentrations (e.g., HG4MB1), suggests that denser cross-linking networks reduce the flexibility and elasticity of the hydrogel matrix, making them more rigid [28].

This result is consistent with the literature, which shows an inverse relationship between cross-linking density and flexibility in hydrogels [29]. Moreover, the viscoelastic properties of the hydrogels exhibited shear-thinning behavior, which is characteristic of hydrogels intended for injectable or flowable applications [30]. The marked reduction in viscosity at higher shear rates, particularly in hydrogels with lower GA concentrations, highlights the potential of these materials for use in dynamic environments where flowability is essential.

Fourier-transform infrared (FT-IR) spectroscopy provided further evidence of the chemical interactions between gelatin, GA, and MB. The characteristic shifts in the amide bands (amide I and II) after cross-linking suggest the formation of Schiff bases between the amino groups of gelatin and the aldehyde groups of GA, a well-established mechanism of cross-linking in gelatin-based hydrogels [31]. Interestingly, the FT-IR spectra of the HGMBs revealed a slight increase in the hydroxyl and amine bands, as well as changes in the amide I band, suggesting that MB may interact with the cross-linking network. This interaction could potentially reduce the formation of Schiff bases, further influencing the hydrogel’s physical properties, as observed in the swelling and mechanical property measurements. These findings align with previous research indicating that the inclusion of small molecules like MB could alter the chemical network of gelatin hydrogels [32].

The DSC results also provide valuable insights into the synthesis and crosslinking behavior of the HGMBs. The thermal properties of gelatin and HG1MB1 show similar trends, with an isomerization and a decomposition transition (Td) observed at 215 °C for gelatin and 226 °C for HG1MB1. The shift in Td to a higher temperature for HG1MB1 indicates that the crosslinked covalent bonds contribute to increased thermal resistance compared to pure gelatin. The absence of a melting transition in the thermograms of all hydrogels suggests that the characteristic crystalline junction zones were likely disrupted during the crosslinking process. In contrast to gelatin, which does not exhibit a defined Tg, the thermograms of HG1MB1–HG3MB1 reveal the appearance of Tg, indicating reduced molecular mobility and a more stable, rigid structure than that of pure gelatin. In the case of HG4MB1, the absence of distinct thermal transitions, including Tg, suggests that the hydrogel structure is highly crosslinked, with extensive covalent bonds between gelatin chains limiting significant molecular motion. These findings align with prior studies on the thermal transitions of gelatin-based hydrogels [32,33], confirming the synthesis pathway and highlighting the impact of GA and MB on the hydrogel’s crosslinking and thermal properties.

The production of ROS by HGMBs was quantified since these species of reactive oxygen play a crucial role in the antimicrobial properties of these hydrogels. Hydrogels with higher MB content, such as HG1MB1, produced the highest levels of ROS, confirming MB’s potential as an effective antimicrobial agent. However, ROS generation decreased with increasing GA concentration, likely due to the reduced availability of MB as the cross-linking density of the hydrogel increased. This trend aligns with previous studies, which have shown that incorporating MB into hydrogels enhances their antimicrobial activity through ROS generation [34].

Given its superior photochemical properties, particularly in ROS production, HG1MB1 was selected for assessing the antimicrobial properties of MB-based photoactive hydrogels. Recent studies have highlighted the critical role of ROS in aPDT, as these molecules are essential for disrupting microbial cell structures and functions [35,36]. Our findings support this, demonstrating that HG1MB1 enhances ROS production, thereby improving the overall antimicrobial efficacy of MB-based aPDT.

The combination of MB with the hydrogel (HG1MB1) significantly enhanced the antimicrobial effect of aPDT, particularly when compared to the control groups without irradiation. This observation corroborates recent research by Glass et al. (2021) [37], which showed that hydrogels can act as effective carriers for photoactive agents, facilitating better light absorption and ROS generation, thus improving microbial inactivation. In our study, control groups that lacked irradiation (L−) did not show significant reductions in microorganism viability, emphasizing the necessity of light activation for effective aPDT. In contrast, the MB-irradiated group (L+) exhibited notable reductions in microbial viability, especially for *S. aureus*, where a log reduction of 3.0 was observed. This also was observed by Usacheva et al., who reported a comparable log reduction of *S. aureus* viability using a photoactive hydrogel, under similar experimental conditions [38]. Importantly, when aPDT was applied using HG1MB1, *S. aureus*, *E. coli*, and *C. albicans* demonstrated significant reductions in viability, with log reductions of 5.40, 4.66, and 3.03, respectively. These results are at least two times greater than those achieved using MB alone, which supports the hypothesis that the hydrogel enhances the photodynamic effect by improving the delivery and retention of MB at the target site [37].

Further analysis of the microorganism-specific responses revealed that *S. aureus* was the most sensitive to aPDT using HG1MB1, followed by *E. coli* and *C. albicans*. The differences in microbial reduction between *E. coli* and *S. aureus*, as well as between *E. coli* and *C. albicans*, were not statistically significant, aligning with previous studies that reported similar susceptibilities of Gram-positive and Gram-negative bacteria to aPDT [39,40]. However, a significant difference in susceptibility to aPDT has been observed between *S. aureus* and *C. albicans*, with fungal species exhibiting greater resistance. This difference is attributed to the robust fungal cell wall structure, composed of chitin and glucans, and enhanced oxidative stress defense mechanisms, which reduce fungal susceptibility to ROS-induced damage [41,42]. Thus, this difference may be attributed to the distinct cellular structures and defense mechanisms between bacteria and fungi, as fungal cell walls are generally more resistant to ROS-induced damage than bacterial membranes.

Overall, our findings demonstrate that MB-based photo-active hydrogels, particularly HG1MB1, show significant promise in enhancing the effectiveness of aPDT against *S. aureus*, *C. albicans*, and *E. coli*. The hydrogel formulation facilitates better delivery of the photosensitizer, leading to increased ROS production, which in turn boosts antimicrobial efficacy. These results are consistent with recent studies highlighting the advantages of hydrogel-based systems in improving the therapeutic potential of aPDT [37]. In contrast, conventional antimicrobial treatments for these pathogens often face limitations such as the development of drug resistance, poor tissue penetration, and adverse side effects. These challenges are especially concerning with organisms like *S. aureus*, *C. albicans*, and *E. coli*, which can rapidly develop resistance to commonly used antibiotics. Our study suggests that MB-based photoactive hydrogel offers a promising alternative, potentially overcoming these limitations by providing a targeted, non-resistant-dependent method for infection treatment.

## 5. Conclusions

This study successfully synthesized and characterized a novel MB-based photoactive hydrogel (HGMBs), through cross-linking gelatin with glutaraldehyde. The HGMBs exhibited excellent thermal stability, predominantly elastic behavior, and non-Newtonian shear-thinning properties, making them highly suitable for future biomedical applications. Swelling and retention studies demonstrated that MB played a key role in the formation of the hydrogel matrix, influencing both cross-linking density and swelling capacity. Rheological analysis revealed a strong, cross-linked network structure, while FT-IR spectroscopy confirmed the formation of Schiff bases between gelatin and GA.

The HGMBs demonstrated efficient ROS generation under light activation, with ROS production directly correlating to MB content, particularly in HGMB1. Consequently, HGMB1 was selected for microbial inhibition studies, where it exhibited strong antimicrobial activity against a variety of microorganisms, including Gram-positive bacteria (*S. aureus*), Gram-negative bacteria (*E. coli*), and fungi (*C. albicans*). These findings highlight HG1MB1 as an effective platform for aPDT, providing a broad-spectrum solution for combating diverse microbial species.

Future studies will focus on evaluating the effectiveness of HG1MB1 in targeting microbial biofilms, as well as its cytotoxicity and in vitro tissue efficacy, to further assess the clinical applicability of this approach in therapeutic settings.

## Figures and Tables

**Table 1:** Swelling behavior, cross-linking properties, and ROS generation of gelatin-based hydrogels (HGs and HGMBs).

Hydrogel	GA 25% (uL/mL)	MB (mg/mL)	Swelling Ratio (%)	M_c_ (g/mol)	V_e_ (Cross-Links/cm^3^)	[MB] in PHBs (mg/g)	ROS
HG1	20	0	8911 ± 446	2.8 × 10^5^ ± 1.4 × 10^4^	2.9 × 10^18^ ± 1.8 × 10^17^	-	-
HG2	40	0	8611 ± 464	2.7 × 10^5^ ± 1.3 × 10^4^	3.0 × 10^18^ ± 1.9 × 10^17^	-	-
HG3	60	0	8599 ± 524	2.7x 10^5^ ± 1.3 × 10^4^	3.1 × 10^18^ ± 1.7 × 10^17^	-	-
HG4	80	0	9067 ± 498	2.9 × 10^5^± 1.8 × 10^4^	2.8 × 10^18^ ± 1.5 × 10^17^	-	-
HG1MB1	20	1	53,581 ± 3482	5.6 × 10^6^ ± 3.1 × 10^5^	1.5 × 10^17^ ± 8.9 × 10^15^	0.416 ± 0.026	1.7 × 10^6^ ± 8.9 × 10^6^
HG2MB1	40	1	44,180 ± 2297	4.1 × 10^6^ ± 2.5 × 10^5^	2.0 × 10^17^ ± 1.1 × 10^16^	0.403 ± 0.023	1.5 × 10^6^ ± 8.0 × 10^6^
HG3MB1	60	1	44,115 ± 2470	4.1 × 10^6^ ± 2.2 × 10^5^	2.0 × 10^17^± 1.0 × 10^16^	0.352 ± 0.019	1.4 × 10^6^ ± 7.6 × 10^6^
HG4MB1	80	1	36,425 ± 1821	3.0 × 10^6^ ± 1.9 × 10^5^	2.8 × 10^17^ ± 1.5 × 10^16^	0.298 ± 0.016	1.1 × 10^6^ ± 5.7 × 10^6^

**Table 2:** Cross-linking density values calculated from swelling and rheological data.

Hydrogel	GA 25% (µL/mL)	V_e_ (mol/cm^3^)	n_e_ (mol/cm^3^)
HG1MB1	20	2.41 × 10^7^ ± 1.48 × 10^8^	1.38 × 10^9^ ± 4.66 × 10^10^
HG2MB1	40	3.32 × 10^7^ ± 1.83 × 10^8^	5.63 × 10^10^ ± 1.47 × 10^11^
HG3MB1	60	3.33 × 10^7^ ± 1.66 × 10^8^	7.10 × 10^10^ ± 2021 × 10^11^
HG4MB1	80	4.58 × 10^7^ ± 2.49 × 10^8^	6.01 × 10^10^ ± 5.82 × 10^11^

## Data Availability

The original contributions presented in the study are included in the article, further inquiries can be directed to the corresponding author.
